# C/EBPbeta-2 confers EGF-independent growth and disrupts the normal acinar architecture of human mammary epithelial cells

**DOI:** 10.1186/1476-4598-4-43

**Published:** 2005-12-21

**Authors:** Linda Bundy, Sam Wells, Linda Sealy

**Affiliations:** 1 Department of Molecular Physiology & Biophysics, Vanderbilt University School of Medicine; Nashville, TN 37232, USA; 2 to whom inquires should be addressed

## Abstract

**Background:**

The transcription factor, C/EBPbeta, is a key regulator of growth and differentiation in the mammary gland. There are three different protein isoforms of C/EBPbeta. C/EBPbeta-1 and -2 are transactivators, and differ by only 23 N-terminal amino acids present in beta-1 only. C/EBPbeta-3 (LIP) lacks the transactivation domain and represses transcription. Elevated C/EBPbeta-2 expression causes MCF10A normal human mammary epithelial cells to become transformed, undergo an epithelial to mesenchymal transition (EMT), and acquire an invasive phenotype. C/EBPbeta is a downstream transcriptional target of Ras signaling pathways and is required for Ras transformation of some cell types. Ras signaling pathways are activated in mammary epithelial cells by the ErbB receptor tyrosine kinase family. Therefore, we considered whether elevated C/EBPbeta-2 expression would resemble ErbB RTK activation in MCF10A cells.

**Results:**

We show that elevated C/EBPbeta-2 expression confers EGF-independent growth in MCF10A mammary epithelial cells. However, MCF10A cells expressing C/EBPbeta-3 are not EGF-independent, and high C/EBPbeta-3 or LIP expression is incompatible with growth. C/EBPbeta-2 overexpression disrupts the normal acinar architecture of MCF10A cells in basement membrane cultures and induces complex multiacinar structures with filled lumen, similar to the consequences of aberrant ErbB2 activation.

**Conclusion:**

Given the ability of C/EBPbeta-2 to confer EGF-independent growth to mammary epithelial cells as well as its capability for disrupting normal epithelial architecture and causing EMT, it is worth considering whether inhibitors which target ErbB family signaling pathways could be less effective in mammary epithelial cells with elevated nuclear C/EBPbeta-2 expression.

## Introduction

The activation of tyrosine kinase receptors plays an important role in the genesis of breast cancer. From the extensive analysis of many breast tumors it is well established that ErbB tyrosine kinase receptors, in particular ErbB2 and ErbB1 (epidermal growth factor receptor, EGFR), often become constitutively active in breast cancer as a result of overexpression, or in the case of ErbB1, autocrine ligand production or mutation (for reviews see [1-4]). Approximately 25% of invasive breast cancers exhibit ErbB2 gene amplification and the rate of ErbB2 gene amplification or protein overexpression in ductal carcinoma in situ (DCIS) is the same or higher than in invasive cancers [2,3]. EGFR and ErbB2 co-overexpression in breast tumors is associated with resistance to endocrine therapies ([5,2] and references therein). Recognizing the alterations in EGFR family tyrosine kinase function galvanized the development of one of the first approved targeted cancer therapeutics, Herceptin, an antibody inhibitor of ErbB2 (reviewed in [6]). The success of Herceptin and other therapies targeting the ErbB receptor family in the treatment of breast cancer patients has so far been mixed [7,8]. This outcome likely reflects the complexity of ErbB receptor tyrosine kinase (RTK) family signaling and the potential to activate alternative pathways, for example, insulin-like growth factor receptor (IGF-R) signaling ([9] and references therein).

A common feature of receptor tyrosine kinases is that their activation generates tyrosine-phosphorylated recognition motifs for the binding of signaling proteins containing Src homology 2 domains. The ErbB receptor tyrosine kinases transmit proliferative signals to the nucleus via multiple signaling pathways, including the Shc- and/or Grb2-activated Ras-Raf-MAPK pathway and phosphatidylinositol-3-kinase (PI-3 K) pathways (reviewed in [1]). Ultimately RTK signaling modulates the activity of transcription factors within the nucleus, leading to alterations in the program of gene expression within the cell. Transcription factors targeted by the ErbB receptor family are likely to have key roles in controlling the growth and proliferation of epithelial cells, given the essential role which alterations in ErbB receptor function have on oncogenic transformation.

Gene deletion studies have shown that the transcription factor, C/EBPbeta, is in fact just such a master regulator of growth and differentiation of the mammary gland [10,11]. C/EBPbeta is a member of a family of basic-leucine zipper transcription factors that play a decisive role in the function of many cell types (reviewed in [12,13]). The mammary epithelial cells (MECs) of C/EBPbeta null mice fail to proliferate in response to hormonal signals at puberty and during pregnancy, and the MECs fail to differentiate in response to lactation-specific hormones resulting in failure to lactate upon parturition [10,11]. Using BrdU labeling, Robinson et al. [10] demonstrated that increased epithelial cell proliferation in early pregnancy and proliferation at late pregnancy stages were strongly impaired in the absence of C/EBPbeta. Furthermore, no expression of β-casein or WAP mRNA was detected in mammary tissue from mutant mice late in pregnancy. Thus, these mice display a dual phenotype: a defect in mammary epithelial cell proliferation in response to hormonal stimulation at puberty or pregnancy, as well as a defect in epithelial cell differentiation in response to lactation-specific hormones.

The production of multiple isoforms of C/EBPbeta in cells may be one mechanism by which a single transcription factor can regulate both differentiation and proliferation. Three isoforms of C/EBPbeta can be produced in cells via alternative translation initiation at 3 in frame methionines [14,15]. C/EBPbeta-1 and -2 (also called LAP* and LAP, respectively) are transactivators, and differ by just 23 N-terminal amino acids present in beta-1 but not beta-2 (value for human, chimp and bovine proteins, 21 aa in mouse, rat and chicken proteins and 22 aa in Xenopus laevis). C/EBPbeta-3 (also called LIP) lacks the N-terminal half of C/EBPbeta, including the transactivation domain, and therefore represses transcription.

Although many initially assumed that C/EBPbeta-1 and -2 would be functionally redundant transactivators because of their extensive similarity, this does not appear to be the case. Only C/EBPbeta-1 (LAP* or LAP1) interacts with the Swi/Snf chromatin-remodeling complex, an interaction that requires the N-terminal amino acids absent from C/EBPbeta-2 [16]. We have also recently found that C/EBPbeta-1, but not -2, is sumoylated at lysine 173, a modification that may uniquely direct C/EBPbeta-1 to specific subnuclear locations and/or protein partners [17]. The different expression patterns of C/EBPbeta-1 and -2 also suggest non-overlapping functional roles for the two transactivators. C/EBPbeta-1 is the only isoform present in normal tissue from reduction mammoplasty [18]. However, 70% of invasive surgical primary breast tumor samples have acquired a high level of C/EBPbeta-2 expression, and C/EBPbeta-2 is the only transactivator isoform expressed in breast cancer cell lines [18]. Recently, we have shown that MCF10A normal human mammary epithelial cells, engineered to selectively overexpress C/EBPbeta-2, become transformed, undergo an epithelial to mesenchymal transition (EMT), and acquire an invasive phenotype [19]. MCF10A C/EBPbeta-2 cells are anchorage-independent, form foci in soft agar, show loss of junctional E cadherin localization, exhibit cytoskeletal reorganization with actin stress fibers typical of motile fibroblasts, express vimentin, and are invasive in vitro [19].

C/EBPbeta is known to be phosphorylated by numerous kinases [20-24] and thus is targeted by many signaling pathways including those that activate the ERK and RSK kinases. In fact, Zhu et al. [25] have recently reported that C/EBPbeta is an essential mediator of skin tumorigenesis involving oncogenic Ras signaling. C/EBPβ null mice are completely refractory to carcinogen-induced skin tumors involving mutant Ras. Because the ERK and RSK kinases are downstream targets of the Shc- and/or Grb2-activated Ras-Raf-MAPK pathways and phosphatidylinositol-3-kinase (PI-3 K) pathways activated by ErbB receptor tyrosine kinases, it is possible that C/EBPbeta-2 is a downstream target of ErbB receptor tyrosine kinase signaling in mammary epithelial cells. Here we demonstrate that MCF10A cells, which are normally dependent on EGF for growth, no longer require EGF when C/EBPbeta-2 is overexpressed. It has been argued that C/EBPbeta-3 or LIP is an important oncogenic mediator of EGF signaling [26,27]. However, we find that LIP expression in MCF10A cells does not confer EGF-independent growth. In fact, overexpression of LIP is incompatible with growth and the LIP-positive cells quickly disappear from the population.

MCF10A cells, although immortal, are often used as a model of normal glandular epithelium because they form growth-arrested three-dimensional acinar structures when grown in basement membrane cultures [28-30]. These structures resemble the individual acinar units of polarized epithelial cells surrounding a hollow lumen that form the terminal ductal lobular units in the adult breast. It is now well established that maintaining this well-ordered epithelial architecture is crucial to maintaining a differentiated state and the control of cell proliferation [31]. The carcinogenic process that disrupts the hollow, polarized architecture is mimicked by the expression of activated ErbB2 receptors in MCF10A cells [29]. Activation of ErbB2 signaling in MCF10A acini reinitiates proliferation and induces complex multiacinar structures with filled lumen [29,49]. We show that overexpression of C/EBPbeta-2 results in a strikingly similar disruption in the 3D architecture of MCF10A cells in basement membrane cultures. Our data indicate that elevated C/EBPbeta-2 has many of the same consequences as aberrant ErbB signaling in mammary epithelial cells.

## Results

### C/EBPbeta-2 confers EGF-independent growth

We have previously described [19] the development of MCF10A cells overexpressing C/EBPbeta-2 via infection with a chimeric retrovirus encoding C/EBPbeta-2 (LZRS-His C/EBPbeta-2). Expression of only the C/EBPbeta-2 isoform was achieved by deletion of amino acids 1–21 of the rat protein. It is important to note that C/EBPbeta-3 cannot be translated from our C/EBPbeta-2 construct. This is because expression of C/EBPbeta-3 depends upon a small (9aa) evolutionarily conserved alternative open reading frame (ORF) located **before **the second in frame ATG of C/EBPbeta [14,15] that is deleted with the N-terminal aa 1–21 truncation. Loss of this 9aa ORF is sufficient to eliminate C/EBPbeta-3 expression [15].

As previously described, viable, non-adherent cells began to accumulate in C/EBPbeta-2 overexpressing cultures within 3 days post infection [19]. These non-adherent cells can be subcultured and provide a non-clonal population of cells overexpressing C/EBPbeta-2. To provide a control population of cells, we also infected MCF10A cells with a beta-galactosidase encoding retrovirus (LZRS-beta-gal). Due to the high titer of the LZRS retroviral system, we were able to obtain >95% infection of MCF10A cells with the LZRS-beta-gal virus.

**Figure 1:**
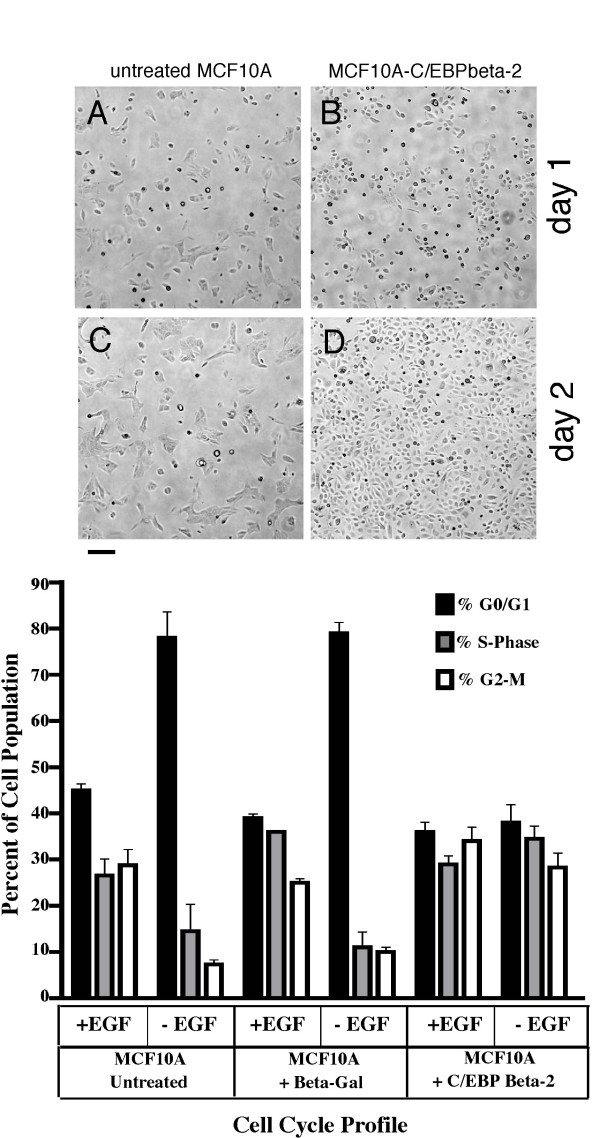
*Exogenous C/EBPbeta-2 expression confers EGF-independent growth in MCF10As*. Micrographs of untreated (panels A and C) and C/EBPbeta-2 overexpressing (panels B and D) MCF10A cultures are shown one (panels A and B) and two (panels C and D) days after growth in medium lacking EGF. Cells were plated on day 0 at 5 × 10^5 ^per 60 mm dish and the same location was imaged on both days. The bar represents 600 microns. Cell cycle profiles of control MCF10A(untreated), MCF10A cells infected with LZRS-Lac-Z virus (+ Beta-Gal), and MCF10A cells infected with LZRS-His-C/EBPbeta-2 virus (+ C/EBPbeta-2) were determined after growth in the presence or absence of EGF for 3 days. Results are shown as the mean of 4 separate experiments. Error bars indicate ± standard error of the mean (SEM). Solid black bars indicate the percentage of cells present in G0/G1, gray-filled bars the percentage in S-phase, and white-filled bars the percentage in G2/M. When compared to control MCF10A and MCF10A + Beta-Gal cell populations grown in the absence of EGF, the cell cycle distribution of MCF10A + C/EBPbeta-2 grown under identical conditions was determined to be statistically significant (p < 0.0001 for G0/G1 and G2/M and p < 0.0085 for S-phase). In contrast, there was no significant difference in the cell cycle distribution of MCF10A + C/EBPbeta-2 cells cultured in the absence of EGF compared to any of the MCF10A-based cells grown in the presence of EGF.

Typically, MCF10A cells, which are not transformed, require EGF in the culture medium for growth. To analyze the effect of C/EBPbeta-2 expression on EGF dependence, we plated uninfected MCF10A cells or C/EBPbeta-2-MCF10A cells at equal densities (5 × 10^5 ^per p60) in the presence or absence of EGF on day 0. (Note that both cultures are maintained in 5% horse serum, only the EGF has been removed). Photomicrographs of the cultures 1 or 2 days later are shown in Figure 1A. Uninfected MCF10A cells (panels A and C) are still very sparse after 2 days, remaining essentially at the same density they were originally plated. In contrast, C/EBPbeta-2-MCF10A cells (panels B and D) are able to proliferate in the absence of EGF and the culture is approaching confluence 2 days after plating. To confirm this observation we performed cell cycle analyses on the cultures by FACS. As shown in Figure 1B, a typical culture of MCF10A cells growing logarithmically in the presence of EGF contained 45% of the cells in G0/G1 and 25% or 28% in S phase and G2/M, respectively. Upon removal of EGF, the proportion of cells in G0/G1 increased to 80%, indicating a substantial growth arrest, consistant with the photomicrographs in Figure 1A. Control infected cells expressing beta-gal behaved similarly and were substantially growth arrested upon removal of EGF from the medium. In contrast, there was no significant change in the fraction of C/EBPbeta-2-MCF10A cells in G0/G1 whether or not EGF was present in the growth medium. The proportion of C/EBPbeta-2-MCF10A cells in S or G2/M cells was also not perturbed by EGF withdrawal. Therefore, we conclude that C/EBPbeta-2 expression confers EGF-independent growth onto MCF10A cells. In addition to cell cycle profiles being unchanged, the doubling time of C/EBPbeta-2-MCF10A cells was not different in the presence or absence of EGF (data not shown).

### EGF signaling network in C/EBPbeta-2 expressing cells is still EGF dependent

**Figure 2:**
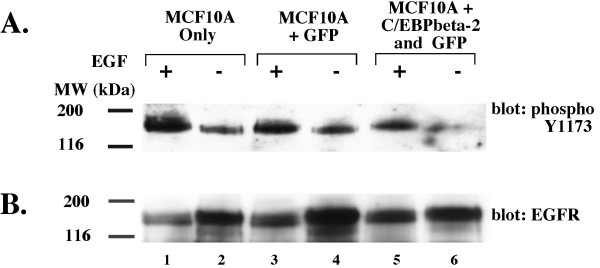
*EGFR is unaltered in C/EBPbeta-2 overexpressing MCF10As*. Uninfected MCF10A cells (lanes 1 & 2), sorted MCF10A cells infected with LZRS-GFP virus (lanes 3 & 4), and subcultured MCF10A cells infected with LZRS-His-C/EBPbeta-2-IRES-GFP virus (lanes 5 & 6) were grown in the presence (lanes 1, 3, and 5) or absence (lanes 2, 4, and 6) of EGF for 3 days. Cell extracts were subjected to 8% SDS PAGE and blotted with an antibody that specifically detect **(A) **phosphorylated tyrosine 1173 (pY1173 EGFR). The same blot was reblotted with an antibody that detects total EGFR for normalization **(B)**.

We considered that C/EBPbeta-2 might lead to the EGF-independent growth of MCF10A cells by one of two general mechanisms. First, C/EBPbeta-2 may be (or may be able to substitute for) a key downstream transcriptional target for EGF signaling in these cells. Once C/EBPbeta-2 is overexpressed, EGF signaling is no longer necessary for growth. Alternatively, C/EBPbeta-2 may not reside in the EGF signaling pathway, but may nonetheless be able to upregulate one or more key components of the pathway, in essence turning on the pathway in the absence of ligand. In the simplest instance, C/EBPbeta-2 could just lead to overexpression of the EGF receptor itself. This mechanism has in fact been reported for the transcription factor, YY1, whose expression in MCF10A cells was recently found to confer EGF-independent growth [50]. To investigate this possibility here, we evaluated the expression level and phosphotyrosine status of the EGF receptor by immunoblotting. We infected MCF10A cells with LZRS-hisC/EBPbeta-2/IRES/GFP retrovirus and selected the floating cells, which are all GFP positive cells with a high level of C/EBPbeta-2 expression. A control population of MCF10A cells expressing GFP only was generated by infecting with an LZRS-IRES-GFP retrovirus and sorting the GFP positive cells by FACS. As shown in Figure 2A, withdrawal of EGF leads to a substantial decline in phosphotyrosine 1173, the major autophosphorylation site of the human EGF receptor [32], in both C/EBPbeta-2-MCF10A cells and control GFP-MCF10A cells or uninfected MCF10A cells. The level of EGFR is also similar among the three MCF10A cell populations; we found no evidence that C/EBPbeta-2 leads to an increase in EGFR expression as seen in Figure 2B. Thus, the EGF-independent growth of C/EBPbeta-2-MCF10A cells is not due to upregulation of the EGF receptor or its phosphotyrosine status.

**Figure 3:**
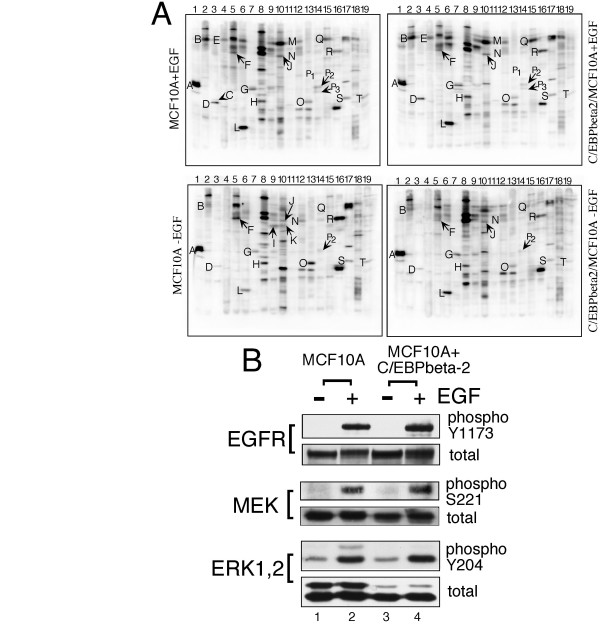
*EGF signaling network remains EGF-dependent in C/EBPbeta-2 overexpressing MCF10As*. **(A) **Kinetworks™ phospho-site screen (KPSS) 2.1 immunoblot analyses of cell extracts prepared from uninfected control MCF10A cells and LZRS-His-C/EBPbeta-2 overexpressing cells (C/EBPbeta2/MCF10A) grown in the presence or absence of EGF for 3 days. Each lane was incubated with a specific mixture of antibodies, and scans of the ECL signals detected with a multi-imager are shown. The identity of specific phosphoylated bands on tyrosine (Y), threonine (T), or Serine (S) residues are as indicated: A SrcY529, B EGFR Y1068, C ERK1 T202/Y204, D ERK2 T185/Y187, E EGFR Y1148, F FAK Y576, G Src Y418, H MEK2 T394, I IGFR Y1162/Y1163, J FAK S910, K Insulin R Y972, L CDK-1 T14/Y15, M IRS1 Y1179, N FAK Y577, O MEK1 S298, P1 Shc Y239/240 (66), P2 Shc Y239/240 (52), P3 Shc Y239/240 (46), Q ErbB2 1139, R FAK S722, S MEK1 T292, T MEK1 T386. **(B) **Immunoblot analyses of whole cell extracts prepared from uninfected MCF10A cells (lanes 1 & 2), or MCF10A cells infected with LZRS-His-C/EBPbeta-2 virus (lanes 3 & 4) which were deprived of EGF in medium containing 0.5% serum for 45 hrs (lanes 1 & 3) and then stimulated with EGF (20 ng/ml) for 30 min (lanes 2 & 4). Protein samples were subjected to 8% SDS PAGE and blotted with an antibodies that specifically detect EGFR phosphorylated on tyrosine 1173, MEK1/2 phosphorylated on serine 221, or ERK1/2 phosphorylated on tyrosine 204 as well antibodies that detect total EGFR, total MEK1/2 or total ERK1/2 respectively.

To gain a broader profile of the phosphorylation status of additional signaling molecules, we analyzed the phosphorylation state of a number of proteins in the ErbB signaling cascade utilizing the immunoblot array technology of Kinetworks (Kinexus Bioinformatics Corp, Vancouver, British Columbia. Note: Because this was not a custom screen, the phosphosites monitored are not limited to EGF signaling). As shown in Figure 3A and presented quantitatively in Table 1, when normal MCF10A cells are grown in the presence of EGF, a number of phosphoprotein components of the EGF signaling network are present as expected, including Shc (Y239/Y240) and ERK2 (T185/Y187) as well as phosphoErbB2 (Y1139). Two additional phosphotyrosine sites in the EGF receptor were present in this screen, Y1148, a major binding site of Shc [33] and Y1068, a binding site for Grb2 [34]. Withdrawal of EGF from the MCF10A cells for 3 days resulted in a substantial decline in both phosphoY1148 and Y1068, similar to that observed for Y1173 in the EGF receptor in Figure 3. PhosphoY1139 in ErbB2 is also strongly reduced in the absence of EGF as are the Shc and ERK2 phosphorylation sites profiled in this assay. These declines are typical of what would be expected from the growth-arrested MCF10A cells in the absence of EGF signaling. Remarkably, although very similar declines in these phosphoprotein sites in the EGF signaling pathway were observed, MCF10A cells expressing C/EBPbeta-2 continue to proliferative without effect.

**Table 1:** Summary of Kinetworks KPSS2.1 Phosphosite Screen.

Lane	Band	Protein /Epitope	Normalized CPM			
			MCF10A + EGF	MCF10A	MCF10A + EGF + C/EBPbeta-2	MCF10A +C/EBPbeta-2

2	B	EGFR Y1068 ^a^	2392	1354	3143	1468
4	E	EGFR Y1148 ^a^	922	N/D	1759	N/D
15	Q	ErbB2 Y1139 ^a^	1641	324	3040	421
14	P1	Shc Y239/Y240 (66) ^a^	223	N/D	79	N/D
14	P2	Shc Y239/Y240 (52) ^a^	575	331	365	231
14	P3	Shc Y239/Y240 (46) ^a^	209	N/D	181	N/D
8	H	MEK2 T394 ^b^	1376	1710	1964	1245
13	O	MEK1 S298 ^a^	765	2204	604	786
17	S	MEK1 T292 ^c^	783	785	643	443
19	T	MEK1 T386 ^c^	632	509	289	270
3	C	ERK1 T202/Y204 ^a^	125	N/D	N/D	N/D
3	D	ERK2 T185/Y187 ^a^	988	237	1189	282
9	I	IGF1R Y1162/Y1163 ^a^	N/D	795	N/D	N/D
10	K	Insulin R Y972 ^a^	N/D	431	N/D	N/D
12	M	IRS1 Y1179 ^a^	1006	N/D	1389	N/D
5	F	FAK Y576 ^a^	653	1569	526	323
10	J	FAK S910 ^b^	978	393	940	730
12	N	FAK Y577 ^a^	485	768	754	727
16	R	FAK S722 ^c^	955	5996	1734	1360
7	G	Src Y418 ^a^	1258	842	1283	1138
1	A	Src Y529^c^	6954	8349	5425	8944
5	L	CDK1 T14/Y15^c^	4591	1106	5284	2514

a, b, and c indicate a(n) stimulatory, unknown, or inhibitory effect on protein function, respectively.

To confirm and extend the Kinexus phosphosite screen, we performed immunoblots with individual antibodies in the EGF signaling pathway from EGFR to ERK1/2. In this set of experiments we prepared extracts from cells that had been deprived of EGF for 48 hrs (Fig. 3B, lanes 1&3) or subsequently stimulated with EGF for 30 min (Fig. 3B, lanes 2&4). Once again we find that C/EBPbeta-2 expression does not change the total level of EGFR and both cell types respond normally to EGF stimulation with strong induction of tyrosine phosphorylation of the receptor on Y1173 as shown. Although three phosphoMEK sites were included in the Kinexus screen, the two serine residues at positions 217 and 221 activated by Raf kinases were not. We therefore probed for serine221 phosphorylation upon EGF stimulation as shown in Fig. 3B. Phosphorylation of serine221 in MEK1/2 was strongly induced in MCF10A cells after EGF treatment (Fig. 3B, lanes 1&2) and expression of C/EBPbeta-2 in these cells did not alter this response (Fig. 3B, lanes 3&4). We also directly confirmed the activation of ERK1/2 seen in the Kinexus phosphoscreen by monitoring tyrosine 204 phosphorylation, one of two targets for MEK1/2 phosphorylation of ERK1,2. As shown in Fig. 3B, EGF stimulation of MCF10A cells resulted in a substantial increase in Y204 phosphorylation of p42ERK2 and a weaker, but detectable increase in p44ERK1 Y204 phosphorylation. MCF10A cells expressing C/EBPbeta-2 exhibited the same increase in p42ERK2 Y204 phosphorylation, but phosphoY204 was undetectable in p44ERK1. The results of Fig. 3B coincide closely with the Kinexus phosphoscreen for ERK1/1 (see Table 1, bands C&D). Interestingly, analysis of total ERK1 and 2 in the MCF10A-C/EBPbeta -2 cells suggests that the lack of phosphoERK1 may be due to a constitutively lower level of ERK1 in the MCF10A-C/EBPbeta-2 cells (see Fig. 3B, lanes 3,4). We do not know why C/EBPbeta-2 expression would result in a decline in ERK1, although the cells obviously continue to proliferate despite the deficit. In any case, we conclude that elevated C/EBPbeta-2 expression does not activate EGF signaling pathways; rather, a high level of this transcription factor bypasses reliance on these signaling pathways.

### C/EBPbeta-3 or LIP does not confer EGF-independent growth

**Figure 4:**
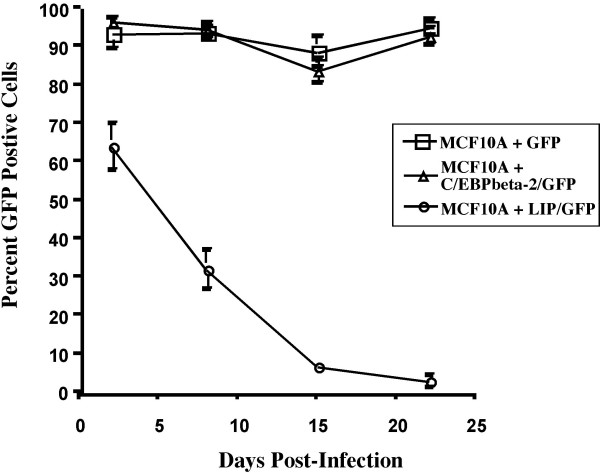
*LIP overexpression is incompatible with cell growth*. MCF10A cells were infected with LZRS-GFP virus (□), LZRS-HisC/EBPbeta-2-IRES-GFP virus (Δ) or LZRS-HisLIP-IRES-GFP virus (○) and monitored for maintainence of GFP expression for 3 weeks post infection. Results are shown as the mean percentage of GFP cells observed in 4–7 randomly selected microscopic fields per time point ± SEM. The loss of GFP expression observed in the LIP/GFP infected culture was determined to be very significant with a p value < 0.0001. Statistical analysis of the linear trend for the GFP only and C/EBPbeta-2/GFP infected cell populations was not significant (p values > 0.05).

Although C/EPBbeta-2 can induce EMT in MCF10A cells in culture [19], it has also been argued that the repressor isoform, C/EBPbeta-3 or LIP, is predominantly expressed during proliferative cellular responses and is associated with aggressive tumors [26,27]. We therefore asked whether C/EBPbeta-3 could also confer EGF-independent growth upon MCF10A cells. MCF10A cells were infected with a chimeric LZRS retrovirus selectively encoding LIP, LZRS-LIP-IRES-GFP, or a control retrovirus expressing GFP only (LZRS-IRES-GFP). We also infected MCF10A cells with LZRS-C/EBPbeta-2-IRES-GFP for direct comparison. We initially analyzed the populations for GFP positive cells 3 days postinfection in normal growth medium containing EGF. Both the GFP only and C/EBPbeta-2 expressing cells were >90% GFP positive, reflecting the high titer of the LZRS system, whereas the LIP-expressing cells were 65% GFP positive (Figure 4). At day 5 postinfection the percent of GFP positive LIP-expressing cells had dropped to 50% and 10 days later (day 15 pi) it was down to <10%. Meanwhile the GFP positive C/EBPbeta-2 expressing cells or GFP only cells remained relatively constant at >90%. It is clear from Figure 4 that a high level of LIP expression is incompatible with continued proliferation, even in the presence of normal growth medium containing EGF.

**Figure 5:**
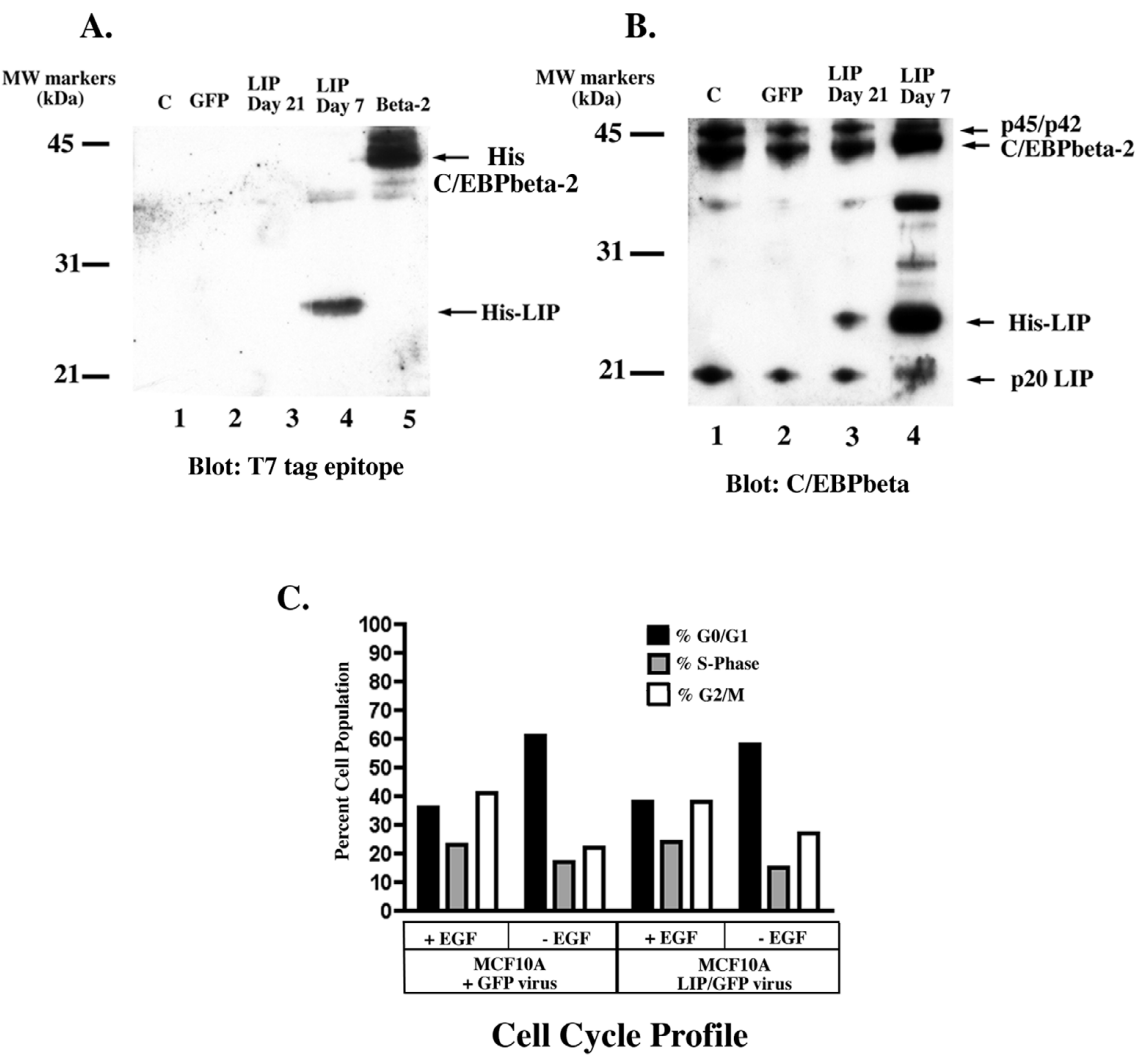
*Exogenous LIP is lost in growing MCF10A cultures*. Cell extracts were prepared from uninfected MCF10A cells (lane 1), GFP positive cells sorted by flow cytometry (lanes 2, 3, & 4) infected with either LZRS-GFP virus (lane 2) or LZRS-HisLIP-IRES-GFP virus (lanes 3 & 4). The sorted cells were placed in culture and allowed to expand. Extracts were analyzed from cell samples collected at 7 days (lane 4) or 21 days (lanes 2,3) post sort. Cell extracts were also prepared from a floating subculture of MCF10A cells infected with LZRS-His-C/EBPbeta-2-IRES-GFP (lane 5) 12 days after the C/EBPbeta-2/GFP overexpressing subculture was established. Western analyses of the extracts is shown probed with anti-T7 epitope tag antibody **(A) **or anti-C/EBPbeta C-terminal antibody **(B)**.

We next attempted to generate a population of LIP-expressing cells by infecting MCF10A cells with LZRS-LIP-IRES-GFP and then immediately sorting the GFP-positive cells by FACS. We reasoned that, after sorting, selecting for cell growth would establish a population of cells expressing LIP at a level that was at least compatible with continued proliferation. We examined the level of LIP expression at various intervals after sorting as shown in the immunoblot in Figure 5. 1 week after sort (the earliest time we had enough cells to prepare a whole cell extract) T7 tag antibody detected strong hisLIP expression (hisLIP migrates at approx. 25 kd, rather than 20 kd, due to the epitope tag), although not quite as robust as we observe with his-taggedC/EBPbeta-2 expressing cells shown in Fig. 5A, lane 5 for comparison. However, when we reexamined the LIP-MCF10A cells at 21 days post sort, we could no longer detect hisLIP expression (Fig. 5A, lane 3) with the T7tag antibody at this exposure level. We conclude that continued proliferation of MCF10A cells in culture is permissible only when LIP expression is drastically reduced. When examined with anti-C/EBPbeta antibody (Figure 5B), we found that the level of exogenous LIP was reduced to approximately that of the endogenous LIP expression in MCF10A cells 21 days post sort. However, the cells expressing a low level of hisLIP were still dependent on EGF for growth (Figure 5C). Thus, not only does LIP not confer EGF-independent growth, but it is also strongly growth inhibitory at the high expression level shown in Figure 5. It may seem surprising then that a high level of LIP has been associated with aggressive tumors. It is well established that the longer isoforms of C/EBPbeta are highly susceptible to proteolysis, giving rise to a relatively protease resistant core that is the same size as LIP [35,36]. From our data it is likely that aggressive breast tumors initially may have contained high levels of C/EBPbeta-2, which was converted to LIP during handling and/or extract preparation.

### C/EBPbeta-2 expression disrupts the acinar architecture of MCF10A cells in basement membrane cultures

**Figure 6:**
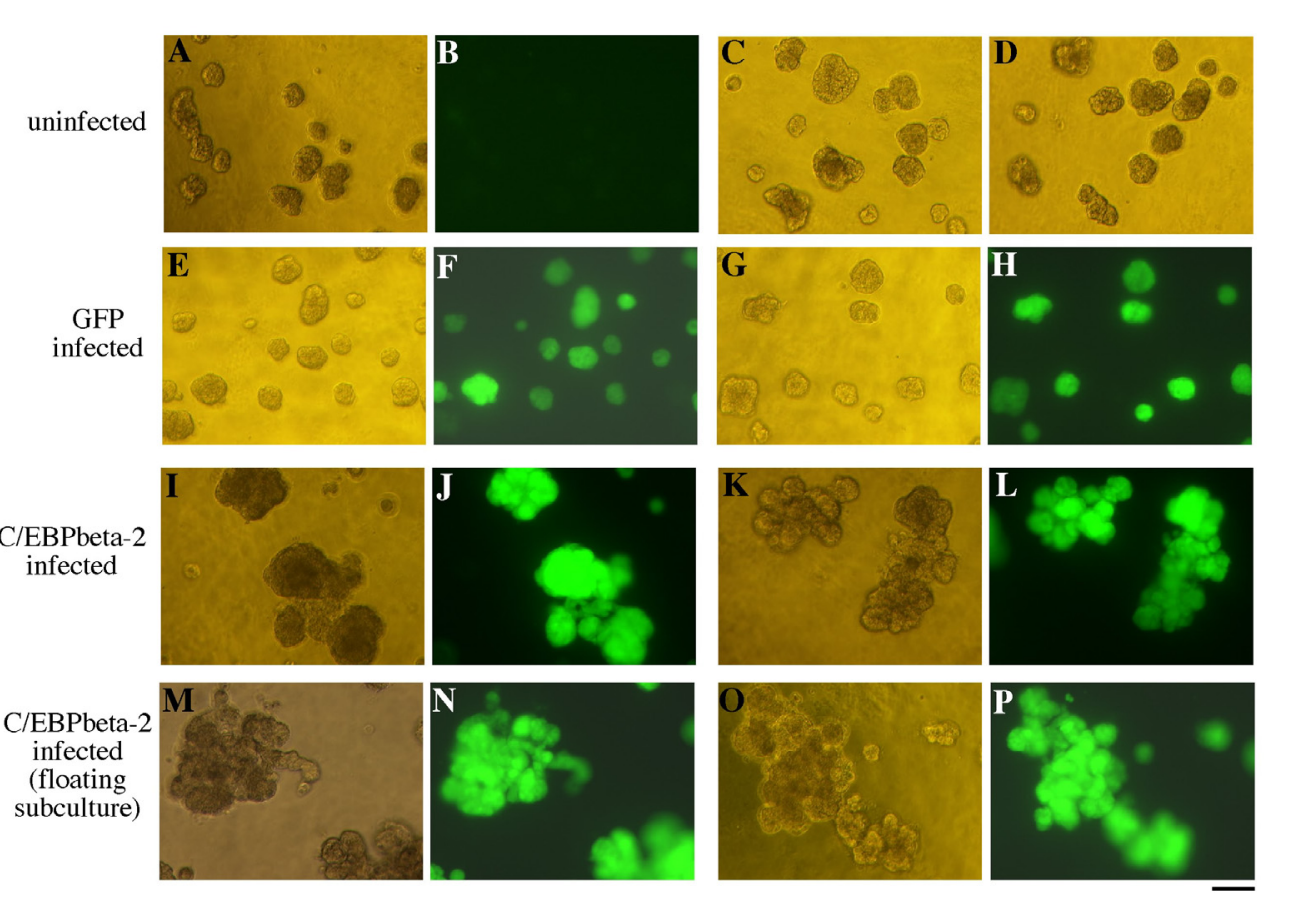
*Morphology of MCF10A acini cultured on Matrigel basement membrane*. Untreated MCF10A cells (A–D), MCF10A cells infected with LZRS-GFP virus (E–H), unselected MCF10A cells infected with LZRS-His-C/EBPbeta-2-IRES-GFP virus (I–L), and a floating subculture of MCF10A cells derived from the aforementioned LZRS-His-C/EBPbeta-2-IRES-GFP culture (M–P) were overlayed on Matrigel and cultured for 9 days. Light micrographs (A, C, D, E, G, I, K, M, & O) and the corresponding flurorescent images are provided to the left (B, F, H, J, L, N, & P). Since untreated MCF10A cells do not express GFP, only one fluorescent image (B) is provided to demonstrate background fluorescence. All cells were cultured at the same passage number and imaged at the same magnification. Bar represents 100 microns.

ErbB2 expression in MCF10A cells has recently been used to model the events in cancer progression that lead to the destruction of the normal acinar architecture of glandular epithelium within the mammary gland [29,30]. Activation of a chimeric ErbB2 receptor using a dimerizing ligand in MCF10A cells induces proliferation and mult-acini formation when the cells are cultured in basement membrane gels. We have used the matrigel overlay culture techniques of Debnath et al. [49] to examine the three dimensional growth of MCF10A cells expressing C/EBPbeta-2 in basement membrane cultures. The morphology of uninfected MCF10A cells, or MCF10A cells infected with GFP only virus or C/EBPbeta-2-IRES-GFP virus are shown in Figure 6. Uninfected MCF10A cells (A-D) form compact acinar structures, which (as will be shown later by confocal microscopy) consist of a single layer of polarized epithelial cells surrounding a hollow lumen. Similar morphology is observed in the MCF10A-GFP only cells. In contrast, cells expressing C/EBPbeta-2 (panels I-L) form large, irregular, multiacinar structures, very similar in appearance to MCF10A cells with activated ErbB2 signaling. MCF10A-C/EBPbeta-2 cells previously selected for anchorage independent growth (panels M-P) also display the irregular, multiacinar phenotype in 3D basement membrane cultures.

**Figure 7:**
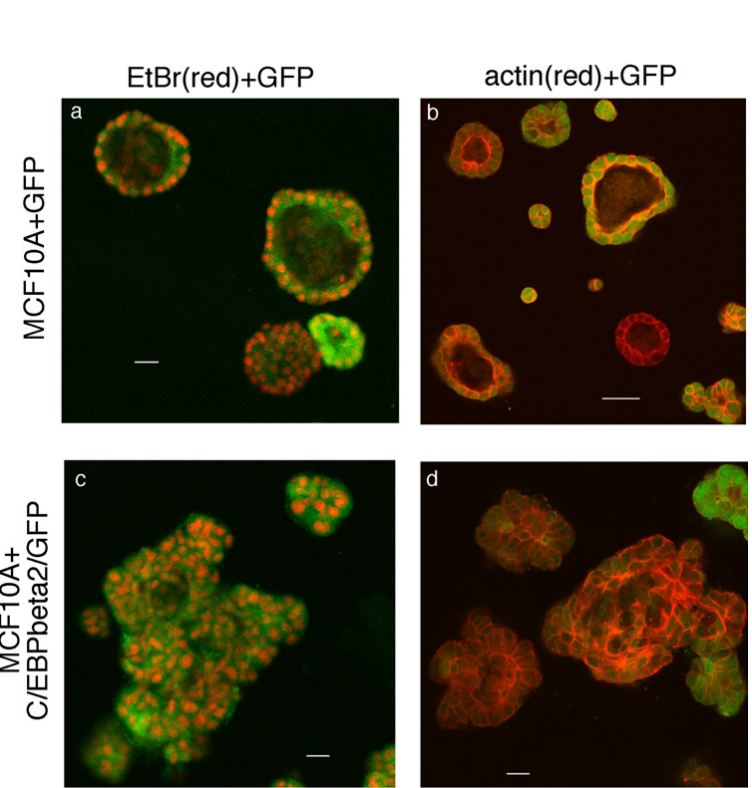
*C/EBPbeta-2 overexpression disrupts three-dimensional acinar structure of MCF10A cells*. MCF10A cells were infected with LZRS-GFP (A & B) or LZRS-His-C/EBPbeta-2-IRES-GFP virus (C & D), overlayed atop a layer of matrigel, and allowed to grow. After 19 days in culture, cells were fixed and stained with ethidium homodimer dye (A & C) to visualize nuclei (shown in red) or Alexa 594 cojugated phalloidin dye (B & D) to visualize f-actin distribution (shown in red). The GFP expression (shown in green) indicates infected cells (A–D). Images were analyzed by confocal microscopy and represent the equitorial cross sections of the acini. Bars represent 22.5 microns.

**Figure 8:**
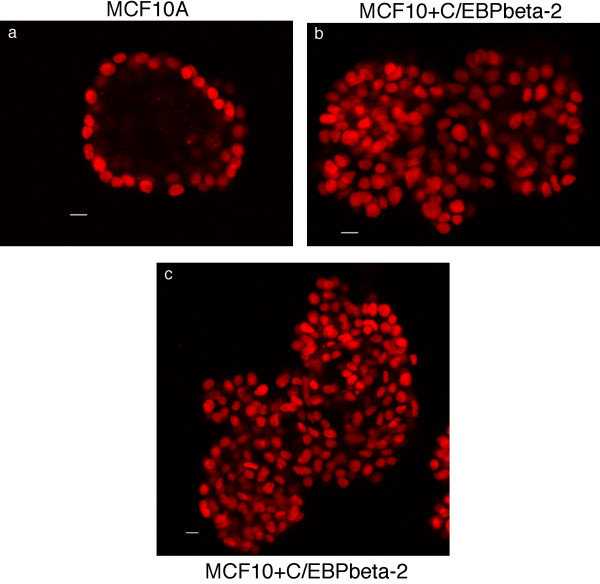
*Ectopic expression of C/EBPbeta-2 disrupts lumen formation*. Additional equitorial cross sections of typical acinar structure observed in MCF10A + GFP cells (A) compared to MCF10A cells overexpressing C/EBPbeta-2 & GFP (B & C). Acini were fixed after 19 days in culture and their nuclei visualized by ethidium homodimer dye (red). Bars represent 11 microns.

To further examine the multiacinar structures we have imaged them by confocal microscopy. In Figure 7, the structures have been stained with either ethidium bromide homodimer dye (nuclear dye, red fluorescence in panels a and c) or phalloidin (F-actin, red fluorescence in panels b and d). Dual imaging of green fluorescence from GFP present in the cells (after retroviral infection) is also shown in Figure 7. The hollow lumens within the compact acinar structures formed by MCF10A cells are easily visible in panels a and b of Figure 7. In contrast, interior confocal imaging of the irregular, multicellular structures formed by the C/EBPbeta-2-MCF10A cells shows the solid, filled lumens of these structures (panels c and d) This is strikingly apparent in Figure 8, where only the red nuclear fluorescence from EtBR homodimer staining is shown. The compact structure formed by MCF10A cells in panel 8a is comprised of a single layer of well organized epithelial cells surrounding the hollow lumen. Structures formed by the C/EBPbeta-2-MCF10A cells (panels 8b and 8c) have an irregular multi-acinar architecture combined with luminal filling. A complete reconstruction of the structures imaged by confocal microscopy in Figures 7 and 8 are available as movie files in the supplementary material (see additional file 1 for panel 7a, additional file 2 for panel 7b, additional file 3 for panel 7c, and additional file 4 for panel 7d. Likewise see additional file 5 for panel 8a, additional file 6 for panel 8b and additional file 7 for panel 8c.)

## Discussion

C/EBPbeta-2 expression enables mammary epithelial cells to proliferate in the absence of EGF signaling pathways in monolayer cultures and to form irregular, multiacinar structures with filled lumen in three-dimensional basement membrane cultures. Muthuswamy et al. [29] have previously shown that activation of ErbB2, but not ErbB1 (EGFR) via homodimerization of a chimeric receptor with a dimerizing ligand, reinitiates proliferation and induces luminal repopulation in epithelial acini in basement membrane cultures, although dimerization of either receptor is sufficient to promote proliferation of MCF10A cells in monolayer culture in the absence of EGF. In dissecting the requirements for luminal filling, Debnath et al. [49] found that inhibiting apoptosis (by exogenously expressing antiapoptotic Bcl family proteins) or enhancing proliferation (via cyclin D1 or HPV E7 overexpression) alone did not result in luminal filling. However, the lumen was filled when oncogenes that enhance proliferation were coexpressed with those that inhibit apoptosis, or when ErbB2, which induces both activities, was activated. Our results in Figs 6, 7, 8 would suggest that like ErbB2, C/EBPbeta-2 may be able to both enhance proliferation and decrease apoptosis resulting in luminal filling. In this regard, not only do C/EBPbeta knockout mice fail to develop Ras-dependent skin papillomas in response to carcinogens such as dimethylbenzanthracene, but a large increase in apoptotic epidermal cells was observed in carcinogen-treated C/EBPbeta-/- mice compared to wt animals [25]. Thus, Zhu et al. [25] have proposed that C/EBPbeta may promote the survival of transformed keratinocytes. More recently, Wessells et al. [37] have shown that Myc/Raf-transformed macrophages require the transcription factor C/EBPbeta to prevent cell death. Transformed C/EBPbeta-/- macrophages undergo programmed cell death upon withdrawal of exogenous hematopoietic growth factors. Ecotopic expression of C/EBPbeta, but not LIP, restored growth factor independent survival to transformed C/EBPbeta null macrophages. Interestingly, C/EBPbeta-dependent regulation of insulin-like growth factor I (IGF-I) expression was shown to play a critical role in the ability of these myeloid tumor cells to escape apoptosis [37]. Thus, C/EBPbeta-2 inhibition of apoptosis would be consistent with the ability of C/EBPbeta to promote survival in other cell types.

It has been noted that the altered mammary epithelial structures formed upon ErbB2 activation exhibit many of the properties of early-stage epithelial tumors, including a high level of proliferation, filling of the lumen, deposition of a basement membrane, and the lack of invasive properties [38]. MCF10A cells with activated ErbB2 are not anchorage independent, maintain E-cadherin adherens junctions, do not invade basement membrane and are not migratory. However, it has recently been found that TGFbeta can cooperate with activated ErbB2 to induce migration and invasion of MCF10A cells [38]. In contrast to MCF10A cells with activated ErbB2, MCF10A cells expressing C/EBPbeta-2 have undergone an epithelial to mesenchymal transition. The cells show loss of junctional E cadherin localization, exhibit cytoskeletal reorganization with actin stress fibers typical of motile fibroblasts and express vimentin [19]. C/EBPbeta-2-MCF10A cells are anchorage-independent, form foci in soft agar, and are invasive in vitro, at least in the Boyden chamber assay [19]. We do not, however, observe the stellate growth pattern typical of invasive and migratory breast cancer cell lines such as MDA231 when C/EBPbeta-2-MCF10A cells are placed in 3D matrigel cultures (Figs. 6, 7, 8). It would be interesting to determine if TGFbeta treatment of these cultures would cooperate with C/EBPbeta-2 to induce the invasive projections that are the hallmark of highly metastatic breast cancer cell lines in 3D matrigel cultures.

We did not find that C/EBPbeta-2 confers EGF independence simply by increasing the level of EGF receptor or conferring ligand independence to the receptor. When EGF is withdrawn from C/EBPbeta-2-MCF10A cells, there is a loss of phosphotyrosine sites on both ErbB2 and EGFR just as is observed in the parental MCF10A cells. Downstream targets such as phosphorylated Shc, MEK1,2 and ERK2 also decline. Yet, despite the lack of EGF signaling, C/EPBbeta-2-MCF10A cells continued proliferating, with no change in cell cycle profile. Although the majority of phosphorylation sites profiled in the Kinexus screen behaved similarly in the MCF10A or C/EBPbeta-2-MCF10A cells, we did observe a few responses specific to the MCF10A cells. For example, FAK phosphorylation on S722 (band R) was strongly enhanced upon withdrawal of EGF in the MCF10A cells only. However, there is little information on the role of S722 FAK phosphorylation, so this difference is difficult to interpret. MEK phosphorylation on S298 (band O) is also enhanced upon EGF withdrawal from MCF10A cells, while little difference is observed +/- EGF in the C/EBPbeta-2-MCF10A cells. S298 in MEK1 is targeted by p21-activated kinase (PAK) in response to integrin signaling and stimulates the association of MEK1 with ERK [39,40]. Perhaps MCF10A cells, in the absence of EGF signaling, may try to compensate by relying more heavily on integrin-mediated signaling. In contrast, MCF10A-C/EBPbeta-2 cells, which are already anchorage independent, do not show this response. However, a more comprehensive analysis of signaling intermediates would be necessary to confirm this interpretation.

Given the wealth of information that C/EBPbeta can be phosphoryated by terminal kinases in EGF signaling pathways such as ERK2 and RSK [16,21-25], it is certainly plausible that C/EBPbeta-2 could normally be a downstream target of EGF signaling. In general, Ras-dependent phosphorylation of C/EBPbeta on Thr235 by the MAP (ERK) kinases is thought to activate the transactivation capability of C/EBPbeta. Nonetheless, our analysis indicates that EGF signaling is not active in the absence of EGF in both MCF10A and MCF10A-C/EBPbeta-2 cells. Thus, C/EBPbeta-2 must be able to mediate the EGF-independent of growth of MCF10A-C/EBPbeta-2 even in the absence of phosphoryation by kinases such as ERK2. One possibility is that C/EBPbeta-2 is overexpressed to a high level that drives the necessary protein-protein interactions through mass action, rendering phosphorylation at sites like T235 unnecessary. Alternatively, other kinases may substitute for the lack of ERK activity. Clearly, although C/EBPbeta-2 does not activate EGF signaling, we have not ruled out that other, compensating signaling pathways could be activated. It is important to note that this study was not meant to decipher whether EGF signaling normally regulates C/EBPbeta-2 function in MECs and how these mechanisms might be bypassed upon C/EBPbeta-2 overexpression. This is likely to be a complex situation better left to future studies.

We did not find that C/EBPbeta-3 or LIP expression was able to confer EGF-independent growth. In fact a high level of LIP expression was incompatible with continued proliferation in culture, since the initially GFP positive, LIP-expressing cells rapidly disappeared from a mixed culture containing both infected and non-infected cells. When we sorted and deliberately tried to expand the GFP positive, LIP expressing cells, we found that the cell population we obtained (i.e. growth selected) had strongly reduced epitope-tagged LIP expression. It has been argued that the transcription inhibitor LIP isoform is predominantly expressed during proliferative cellular responses and is associated with aggressive tumors. Zahnow et al. [29] have reported that LIP is overexpressed in 23% of infiltrating ductal carcinomas specimens. However, our own study on primary breast tumor samples found that high grade, invasive mammary carcinomas showed significant C/EBP-2 expression, but no LIP was detected in any of the samples [18]. LIP is known to be easily generated by artifactual proteolysis of the larger isoforms. It has also been reported that expression of LIP under the control of the whey acidic promoter in the mouse mammary gland results in the formation of hyperplastic tissue and carcinomas [27]. However, because the LIP transgene was not epitope-tagged in these mice it is not possible to ascertain transgene expression distinguished from any endogenous LIP expression. Moreover, the level of LIP expression (transgene or enodogenous) in the mammary tumors was not actually examined.

The C/EBPbeta transactivator or LAP has long been known for its role in the activation of differentiation-specific genes in hepatocytes, adipocytes and macrophages. Therefore, the consequences of dominant-negative LIP expression were initially thought to be inhibition of the differentiation-promoting functions of the activator, thereby facilitating proliferation. It has more recently been appreciated that C/EBPbeta is a critical regulator of cellular proliferation/survival as well. C/EBPβ-null mice show profound defects in liver regeneration after partial hepatectomy [22,41]. C/EBPbeta is an essential mediator of skin tumorigenesis involving oncogenic Ras signaling [25]; and, C/EBPbeta is essential for oncogenic transformation of myeloid/macrophage-like cells by *myc*and *raf *[37]. Evidence has now accumulated that differentiation-specific gene expression, to the extent that it requires chromatin-remodeling abilities [16], may be carried out by LAP* (C/EBPbeta-1). Our data would suggest that the growth-promoting role of C/EBPbeta is carried out in large measure by C/EBPbeta-2. The effect of LIP expression very likely depends on which of the longer isoforms, C/EBPbeta-1 or -2, is present in the cells. Unfortunately, it can be extremely confusing to identify C/EBPbeta-1 and -2 in immunoblots by their size or mobility, as it is easy to misidentify a phosphorylated, and therefore slower mobility, C/EBPbeta-2, as C/EBPbeta-1. Nonetheless, for normal cells, where C/EBPbeta-1 is the predominant isoform [18], LIP expression may indeed disrupt differentiation-specific gene functions and in doing so contribute to proliferation. However, in cells where C/EBPbeta-2 is the predominant or only transactivator isoform, antagonizing the function of C/EBPbeta-2 by LIP expression leads to the inhibition of cell growth demonstrated here.

Given the ability of C/EBPbeta-2 to confer EGF-independent growth to mammary epithelial cells as well as its capability for disrupting normal epithelial architecture and causing EMT, it is worth considering whether aberrant C/EBPbeta-2 expression could contribute to the resistance of some breast cancers to targeted therapies such as Herceptin. Unlike C/EBPbeta-1 or -3, C/EBPbeta-2 is found in both the cytoplasmic and nuclear compartments of the cell [18,42]. In fact in normal mammary epithelial cells, C/EBPbeta-2 is primarily sequestered in the cytoplasm [18]. Alterations in the cytoplasmic-nuclear trafficking of C/EBPbeta-2 could result in elevated nuclear levels of this transcription factor, as could overt overexpression and an increase in nuclear levels by mass action. Unfortunately it is not possible to quantitative C/EBPbeta-2 expression without resorting to immunoblotting, because all available antibodies to C/EBPbeta-2 would also recognize C/EBPbeta-1. Immunoblotting is not always practical, and the risk of artifactual isoform generation during preparation of cell or tissue lysates is ever present. Improved tools for C/EBPbeta-2 detection are clearly needed to better assess the contribution of this transcription factor to the unregulated growth of mammary epithelial cells during tumorigenesis.

## Materials and methods

### Cell lines

The previously characterized MCF10A human mammary cell line [43,44] was obtained from the American Type Culture Collection (ATCC) in Manassas, VA. Cells were grown in a 1:1 mixture of Dulbecco's modified Eagle medium (DMEM) and Ham's F12 containing 2.5 mM L-glutamine and supplemented with 5% horse serum (Sigma, St. Louis MO), 10 micrograms/ml recombinant human insulin (GIBCO Invitrogen Corp., Grand Island, NY) 0.5 micrograms/ml hydrocortisone, 10 ng/ml epidermal growth factor (EGF), 100 ng/ml cholera toxin, 50 U/ml penicillin, and 50 micrograms/ml streptomycin, as described previously [19]. Photographs of the cells were taken with an Olympus DP12 microscope/digital camara system. The phoenix-ampho packaging cell line developed by GP Nolan (Stanford University, Palo Alto, CA) was obtained from the ATCC and maintained as previously described [19,45].

### Cloning of recombinant retroviral constructs and virus preparation

The control LZRS-LacZ retroviral supernatants were generated by transfection of the phoenix-amphotropic packaging cell line with the hybrid EBV/retroviral construct, pLZRS-LacZ, as described previously [46]. The previously described LZRS-GFP only, LZRS-His-C/EBPbeta-2, and LZRS-His-C/EBPbeta-2-IRES-GFP viral supernatants were generated in a similar manner as described previously [19]. The bicistronic pLZRS-His C/EBPbeta-3-IRES-GFP vector used to generate LZRS-His-C/EBPbeta-3-IRES-GFP retrovirus was constructed in a multi-step protocol. First, rat C/EBPbeta-3 (also known as LIP and p20 C/EBPbeta) was excised from the previously described pRSETA LIP construct [47] with *Bam*HI and *Eco*RI. The resulting 575 bp C/EBPbeta-3 coding sequence was then ligated into the similarly digested pcDNA3.1HisC expression vector (Invitrogen Life Technologies, San Diego, CA). The LZRS-His-C/EBPbeta-3 construct was generated by replacing the LacZ coding sequences of pLZRS-LacZ with the 711 bp His-tagged C/EBPbeta-3 fragment excised from pcDNA3.1 His C/EBPbeta-3 with *Hin*DIII and *Not*I. Next, the His-tagged C/EBPbeta-3 sequences were subcloned into pIRES2-EGFP expression vector (Clontech, Palo Alto, CA). This was accomplished by digesting pLZRS-His C/EBPbeta-3 with *Eco*RI and *Bgl*II, and ligating the resulting 720 bp fragment into pIRES2-EGFP at the *Eco*RI and *Bam*HI sites. Lastly, the bicistronic pLZRS-His-C/EBPbeta3-IRES-GFP construct was generated by replacing the LacZ coding sequences of pLZRS-LacZ with the 2,036 bp His-C/EBPbeta-3-IRES-GFP coding sequences from pHis-C/EBPbeta-3-IRES-GFP at the *Eco*RI and *Not*I restriction sites.

Recombinant, amphotropic retroviral stocks were generated by transfecting the phoenix-ampho packaging cells independently with each of the above-described LZRS-based constructs as described previously [19,46]. Retroviral transduction and the establishment of floating C/EBPbeta-2 over-expressing subcultures were also described previously [19].

### Flow cytometry

DNA cell cycle profiles of sub-confluent (50–60%) cultures were determined by flow cytometry using a BD FACScan (Becton Dickinson, San Jose, CA). Subconfluent cultures of MCF10A cells and their derivatives were deprived of EGF for 72 hours. If the cells reached confluence during this time period (i.e MCF10A+C/EBPbeta-2 cells without EGF or the control cultures with EGF), the cells were trypsinized and replated at an appropriate density to reach 50–60% confluence at the end of the 72 hr period. All cultures were harvested at 72 hrs by trypsinization and pelleting in the presence of 20% fetal bovine serum at 500 × g for 7 minutes. Following quantification on a hemocytometer, approximately 2 × 10^6 ^cells were washed twice in cold phosphate-buffered saline (PBS) and fixed in ice-cold 70% ethanol (ETOH) overnight. The samples were then pelleted at 500 × g for 7 minutes and washed twice with ice-cold PBS. Lastly, the cells were incubated in a staining solution containing 0.1% (v/v) Triton X-100, 2.5 mg/ml RnaseA, 2.0 mg/ml propidium iodide, 1 microM EDTA in 1 × PBS for 30–60 minutes at 4°C in the dark. Data was collected using BD Cellquest software (BD Biosciences Immunocytometry Systems, San Jose, CA), and cell cycle modeling performed using Modfit software (Verity Software House, Topsham, ME). The cell cycle profile of each population was generated from DNA content data collected from between 17,000 to 23,000 separate events.

In addition, fluorescence-activated cell sorting (FACS) was performed on MCF10A cultures infected with either LZRS-GFP only or LZRS-His-C/EBPbeta-3-IRES-GFP virus to generate pure GFP-expressing populations. Infected populations were trypsinized and pelleted in media containing 20% FBS at 500 × g for 7 minutes. The cells were then resuspended in DMEM/F12 media containing 0.5% horse serum and filtered through a sterile 0.95 micron nylon mesh (Small Parts Inc., Miami Lakes, FL) prior to sorting with a BD FACSAria equipped with FACSDiva software (Becton Dickinson, San Jose, CA). GFP-expressing cells were collected under sterile conditions in DMEM/F12 media containing 20% horse serum, 20 mg/ml gentamycin, 200 U/ml penicillin and 200 mg/ml streptomycin. After the sorted populations were expanded in cell culture, whole cell lysates were prepared and analyzed on immunoblots.

### Cellular proliferation assays

MCF10A cells were infected 3 times with LZRS-GFP only, LZRS-His-C/EBPbeta2-IRES-GFP, or LZRS-His-C/EBPbeta-3-IRES-GFP virus and maintained as usual. Day 0 of the time course was designated as the date of the final viral infection. One day before imaging the cultures, approximately 1 × 10^4 ^cells were passed into a 6 cm dish and allowed to grow overnight. The growth media was aspirated and replaced with a layer of PBS immediately before microscopic examination to minimize background fluorescence from the phenol-red containing media. GFP-fluorescence and transmitted light images of the same representative microscopic field were taken with a using a Hamamatsu C5810 color CCD camera with a 10X/0.25 N Plan Ph1 lens and GFP filter set on a Leica DMIRB inverted microscope. The percentage of GFP positive cells present in 4 to 7 randomly selected representative fields was tabulated and used to calculate the mean percentage of GFP positive cells per plate for each timepoint. All fluorescence images were collected under identical conditions.

### Statistics

Statistical analysis of cell cycle profiles was performed using one-way analysis of variance (ANOVA) with Dunnet Mulitple Comparisons post test utilizing Instat 3.0 for Macs (Graphpad Software, San Diego, CA). Results were calculated as the mean ± SE of 4 separate assays. Each assay included modeled cell cycle profiles from a population of between 17,000 to 23,000 cells. For analysis of cellular proliferation, the percentage of GFP positive cells in a sample was determined from overlapping transmitted light and GFP-fluorescent images of the same microscopic field. Results are expressed as the mean percent GFP positive cells ± SE from 4–7 representative microscopic fields per sample and timepoint. The results were plotted as the percentage of GFP positive cells maintained within each population over a period of time. Statistical analysis of the linear trend of each population was analyzed using ANOVA and Instat 3.0 for Macs. In all instances, results with p values <0.01 were considered significant and p values > 0.05 were not.

### Immunoblot analysis

Comparative levels of activated human epidermal growth factor receptor EGFR in specified MCF10A cultures grown with or without epidermal growth factor (EGF) for 72 hours was determined by standard immunoblot analysis as described previously [19]. In brief, whole cell lysates (WCLs) were prepared from 50–75% confluent 10 cm dishes by scraping into a chilled solution containing protease and phosphatase inhibitors as previously described [18]. Relative protein concentrations were determined using Protein Assay Reagent (BioRad Laboratories, Hercules, CA) as per the manufactures' instructions. Equivalent amounts of total protein were loaded and separated on an 8% SDS-PAGE. Proteins were transferred to an Immobilon P filter and processed as described previously [18]. To detect phosphoEGFR (Y1173), the blot was incubated overnight at 4°C in 0.5% NFDM-TBS-T with 1 microgram/ml anti-phospho-EGFR antibody (clone 9H2, Upstate Cell Signaling Solutions, Charlottesville, VA) and detected with goat-anti-mouse horse-radish peroxidase (HRP)-conjugated secondary antibody (Promega, Madison, WI) in 0.5% NFDM-TBS-T. For detection of total EGFR, the blot was stripped using Re-blot Plus mild antibody stripping solution (Chemicon International Inc., Temecula, CA), probed with a 1: 1000 dilution of anti-EGFR antibody (Upstate), and detected with 1:1000 dilution of biotinylated rabbit anti-sheep IgG (Vector Labs, Burlingame, CA) followed by incubation with peroxidase-conjugated streptavidin (Jackson ImmunoResearch Labs, West Grove, PA). For analysis of EGF signaling, WCLs were prepared from specified MCF10A cultures with or without treatment for 30 min with 20 ng/ml EGF after maintaining the cells for 45 hrs in growth medium as described above except containing 0.5% horse serum and no EGF. After analysis by 8% SDS-PAGE, duplicate immunoblots were probed with anti-EGF receptor or anti-phosphoEGFR (Y1173) as described above or anti-ERK-1 (sc-94, Santa Cruz) or anti-phospho ERK (sc-7383, Santa Cruz). Two blots were stripped using Re-blot Plus mild antibody stripping solution and reprobed with anti-MEK-1 (07–641, Upstate) or antiphospho MEK (S221, Cell Signaling) antibodies. Blots were detected with a 1:5000 dilution of the appropriate goat anti-mouse or goat anti-rabbit secondary antibodies (Cell Signaling).

Kinexus Bioinformatics Corporation (51, Vancouver, British Columbia, Canada) performed Kinetworks™ biosource phospho-site screen (KPSS 2.1) western blot analyses on WCLs prepared from MCF10A cultures treated similarly to those described above [48]. In brief, 350 micrograms of WCL protein were separated by SDS-PAGE, transferred to a thin membrane and, using a 20 lane multiblotter apparatus, probed with a mixture of primary antibodies that react with a distinct subset of phospho-specific proteins of distinct molecular mass.

Quantification of the immunoreactive bands on the Kinetworks blots (trace quantity) with ECL detection was performed with a Bio-Rad Fluor S Max Imager and Bio-Rad Quantity One software.

### Three-dimensional overlay growth assays

In order to assess the three-dimensional growth properties of MCF10A cells and their retrovirally transduced derivatives, cells were grown according to the three-dimensional overlay method described previously [30]. In brief, an 8-well chambered slide was coated with a 2 mm thick layer of growth factor reduced Matrigel, a reconstituted basement membrane obtained from BD Discovery Labware (Bedford, MA). A single cell suspension of 5,000 cells per well was seeded atop the solidified layer of Matrigel and then overlayed with Assay Medium (DMEM/F12 containing 2% Horse Serum, 10 micrograms/ml insulin, 0.5 micrograms/ml hydrocortisone, 100 ng/ml cholera toxin) containing 2% Matrigel and 5 ng/ml EGF. Cells were grown in a humdified incubator with 5% CO_2 _and refed with Assay Medium containing 2% Matrigel and 5 ng/ml EGF every 4 days. Transmitted light and epifluorescence micrographs were acquired using a Hamamatsu C5810 color CCD camera with a 10X/0.25 N Plan Ph1 lens and GFP filter set on a Leica DMIRB inverted microscope.

### Immunofluorescence acquisition and image analysis

MCF10A cells and their retrovirally transduced derivatives were cultured on basement membrane as described above. After 19 days in three-dimensional culture, the acinar structures were fixed and immunostained as described previously with minor modifications [30]. Specifically, acini were fixed with 2% paraformaldehyde in 1 × PBS for 20 minutes at RT, permeabilized with PBS containing 0.5% Triton X-100 for 10 minutes at 4°C, and rinsed three times with PBS/Glycine (130 mM NaCl, 7 mM Na_2_HP0_4_, 3.5 mM NaH_2_P0_4_, and 100 mM Glycine) at RT for 10 minutes. The samples were then blocked with 200 microliters per well of IF Buffer (130 mM NaCl, 7 mM NaN3, 0.1% BSA, 0.2% Triton X-100, 0.05% Tween-20) and 10% normal goat serum (Jackson ImmunoResearch Labs) for 2 hours at RT. To visualize nuclei, samples were stained with 1 micromolar final concentration of ethidium homodimer-2 (EthD-2) (Molecular Probes, Eugene OR) for 15 minutes at RT. To visualize F-actin distribution, samples were stained with Alexa 594-conjugated phalloidin (Molecular Probes) using1 unit in 200 microliters of PBS per well for 15 minutes at RT. Samples were then washed twice with 1 × PBS at RT for 10 minutes, the chambers removed, and mounted in fresh Prolong Antifade reagent (Molecular Probes). Confocal images were acquired with an LSM510 confocal microscope using a Plan-Apochromat 20x/0.75 or Fluar 10x/0.5 objective (Carl Zeiss, New York, NY). The 20× lens was used for all images except figure 7C that required the extra working distance of the 10× lens to visualize the thick cell mass. Ethidium homodimer-2 (red) fluorescence was excited at 543 nm and emission detected through an LP585 barrier filter. Alexa 594 phalloidin (red) fluorescence was excited at 543 nm and emission detected through an LP585 barrier filter. GFP (green) fluorescence was excited at 488 nm and emission detected through a BP505–550 barrier filter.

## Authors' contributions

LB performed all of the retroviral infections and sorting of GFP positive cells. LB carried out the growth analyses of the cells with and without EGF, and performed the 3 dimensional matrigel growth assays. LS performed immunoblots of the EGF receptor, EGF signaling intermediates, and C/EBPbeta-3 expression. LB and LS were both involved in the Kinexus profiling. SW performed the confocal image analyses of acinar structures after preparation and staining by LB. LB and LS conceived of the study and participated in its design. LS coordinated the study and drafted the manuscript.

## Supplementary Material

Additional File 1 Six optical sections of acini comprised of MCF10A cells infected with LZRS-GFP virus show the cell nuclei stained with ethidium homodimer-2 (red) and enhanced GFP expression (green). Image planes progress from the surface of the sphere (Supp. 7a1), downward toward the center (Supp. 7a3), to the opposite surface (Supp. 7a6). Sections are imaged every 9 microns. Click here for file

Additional File 2 Eighteen optical sections of acini comprised of MCF10A cells infected with LZRS-GFP virus illustrate f-actin organization stained with Alexa 594 conjugated phalloidin (red) and enhanced GFP expression (green). Image planes progress from the focal plane near the surface (Supp 7b1), downward toward the center (Supp 7b9), to the opposite surface (Suppl 7b18). Sections are imaged every 3 microns. Click here for file

Additional File 3 Eight optical sections of acini comprised of MCF10A cells infected with LZRS-His-C/EBPbeta-2-IRES-GFP virus show the cell nuclei stained with ethidium homodimer-2 (red) and enhanced GFP expression (green). Image planes progress from the surface of the sphere (Supp 7c1), downward toward the center (Supp. 7c4), to the opposite surface (Supp. 7c8). Sections are imaged every 9 microns. Click here for file

Additional File 4 Montage of sixteen optical sections of acini comprised of MCF10A cells infected with LZRS-His C/EBPbeta-2-IRES-GFP virus illustrate the lack of f-actin organization stained with Alexa 594-conjugated phalloidin (red) and enhanced GFP expression (green). Image planes progress from the focal plane near the surface (Supp 7d1), downward toward the center (Supp 7d8), to the opposite surface (Supp 7d16). Sections are imaged every 3 microns. Click here for file

Additional File 5 Montage of eleven optical sections of acini comprised of MCF10A cells infected with LZRS GFP virus show organization of cell nuclei to form a hollow sphere. The nuclei are stained with ethidium homodimer-2 (red). Image planes progress from the focal plane near the surface (Supp 8a1), downward toward the center (Supp 8a5), to the opposite surface (Supp 8a11). The cells express GFP (not shown). Click here for file

Additional File 6 Montage of sixteen optical sections of acini comprised of MCF10A cells infected with LZRS-His-C/EBPbeta-2-IRES-GFP virus show disorganized cell nuclei that fill the mammosphere. The nuclei are stained with ethidium homodimer-2 (red). Image planes progress from the focal plane near the surface (Supp 8b1), downward toward the center (Supp 8b8), to the opposite surface (Supp 8b16). Although not shown, the cells also express GFP. Click here for file

Additional File 7 Montage of twenty optical sections of acini comprised of MCF10A cells infected with LZRS-His-C/EBPbeta-2-IRES-GFP virus show disorganized cell nuclei that fill the mammosphere. The nuclei are stained with ethidium homodimer-2 (red). Image planes progress from the focal plane near the surface (Supp 8c1), downward toward the center (Supp 8c10), to the opposite surface (Supp 8c20). Although not shown, the cells also express GFP. Click here for file
